# Family and social predictors of substance use disorder in Iran: a case-control study

**DOI:** 10.1186/s13011-019-0201-x

**Published:** 2019-05-06

**Authors:** Gholamhossein Shahraki, Zahra Sedaghat, Mohammad Fararouei

**Affiliations:** 1 Social Determinants of Health Research Center, Yasuj University of Medical Sciences, Yasuj, Iran; 2 Research Center for Health Sciences, Department Epidemiology, School of Health, Shiraz University of Medical Sciences, Shiraz, Iran; 3 HIV/AIDs Research Center, Shiraz University of Medical Sciences, Zand Street, Shiraz, 7143854188 Iran

**Keywords:** Substance use disorder, Risk factors, School attainment, Leisure time

## Abstract

**Background:**

The problem of substance use disorder in Iran is of great national concern. The aim of this study was to measure the association between substance use disorder and demographic, social and behavioral factors in Yasuj city, located at southwest of Iran.

**Methods:**

As the second phase of a previously published study, this case-control study was conducted in 2015–2016 on 362 addicted participants and 207 controls (with no history of substance use disorder). The control group was selected from male hospital visitors in Yasuj city during the same period of selection of the case group. The required information was collected via a self-administered questionnaire.

**Results:**

Based on the results of multivariate analysis, significant associations were found between the number of sisters (OR = 0.82, 95%CI = 0.68 to 0.99, *P* = 0.04), history of smoking (OR_yes/no_ = 19.89, 95%CI = 10.02 to 39.49, *P* < 0.001), leisure time activity (OR_with friends/home_ = 8.98, 95%CI = 3.99 to 20.19, *P* < 0.001) and substance use disorder.

**Conclusion:**

This study introduced smoking, number of sisters, education and way of spending leisure time as predictors of substance use disorder. Among these factors, smoking was the most powerful risk factor and spending leisure time with family and having sister were the most powerful preventive factors for substance use disorder.

## Background

Regardless of age and sex, the global prevalence of drug misuse is continuously a major issue in public health [1, 2]. Along with the growing number of users, substance use disorder is becoming a more serious social problem in developed and developing countries [3]. Substance use disorder is also a serious health issue as it causes a wide range of physiological and psychological disorders [4]. It is believed that adverse effects of substance use disorder is more serious among young individuals as over 90% of substance users experience their first use during their adolescence age and later face substance use disorder and its serious adverse effects including depression, suicide, problems in interpersonal relationships and traffic related injuries or deaths [5–7]. Others showed that short and long term health and social effects of using such substances are serious [8, 9]. For example, based on a report from the Iranian minister of internal affairs, substance use disorder is responsible for about 50% of divorces in Iran. According to the results of a study in Iran, the rate of substance use is approximately 15% among young adults and due to the recent economic recession and social changesو including poverty and unemployment, the figure is rising [8]. Indeed, due to methodological difficulties including recognition and reporting of substance use disorder and drug misuse, the related figures are believed to be highly underestimated [10]. The number of substance users was estimated at about 1.2 million (approximately 1.6% of total population) [11]. According to another study conducted in Iran, in different parts of the country, the usage rate of opioid derivatives is between %2.1 to %20 [6]. It is also suggested that use of heroin, a much stronger derivative of opium, is common among substance users in Iran. Indeed, according to Shekarchizadeh et al. more than 40% of drug users use opium [7].

Numerous studies are conducted to identify the contributing factors to substance use disorder and the results came out in favor of a long list of factors including drug availability, unemployment, peer groups, parent’s behavior, parent’s education and individual’s socio-economic status play important roles in substance use disorder [4, 12, 13]. In addition, substance use in one or more family members is considered as an important contributor to the development of substance use disorder in other members of the family [4]. Accordingly, the stronger is the relationship between an individual and their drug user relatives, the higher is the risk of him/his becoming a drug user [4, 14]. It is also shown that a family’s response to a member’s misbehavior, including drug misuse, is another important factor which affects the risk of an individual to become a substance user [9, 15, 16]. Poor family dynamic (including dysfunctional relationship between family members, especially parents) is associated with delinquency and miss behavior among younger members. It seems that culture based families are more successful in preventing delinquency among their members [17]. Studies, which are conducted on family dynamics in Iran, introduced a strong tide between family relationship, social values and religion [18]. It is possible that parents of culture-based families have a tighter supervision on their children’s behavior. However, a fast cultural transition from culture-based families to more modernized families especially in younger Iranian couples is reported [18]. Friends and individual’s friendship networks are also suggested to be major players in delinquency and drug use [12]. In addition to the above family and social factors, previous studies suggested that smoking has a highly significant association with substance use [12, 19]. However, it is not clear whether the association is causal, and if so, what is the direction of the association (is smoking a risk factor for addiction or vice versa).

The problem of substance use disorder in Iran is of great national concern [7]. As the result, understanding the etiology of substance use disorder is crucial for the government in order to control the social, cultural and economic damages that substance use disorder causes. The increasing number of people suffering from substance use disorder suggests lack of knowledge about the effective contributors and preventive factors of substance use disorder, the most common harmful but preventable behavior in human population [10]. Based on a multidimensional model of delinquency [20], the aim of this study was to measure the association between substance use disorder and some demographic, school and behavioral factors in Yasuj, Iran. The results may be used to predict substance use disorder years before the disorder occurs.

## Methods

### Settings

This case-control study was conducted in 2015–2016 as the second phase of a previous study on substance user participants (*n* = 362) [12] and a group of controls, participants with no history of substance use disorder (*n* = 207), in Yasuj. The city is the capital of Kohgiluyeh and Boyer-Ahmad, a small province located at southwestern part of Iran. Yasuj has about 134,000 population. However, the number of addicted individuals in Yasuj is un-known. This is because substance use is a serious crime in Iran and substance users are a hidden community.

### Selection of cases

The participants in the case group (substance users) were defined as those individuals who were using drugs on a daily basis. Substance users were selected via snowball sampling among males aged between 18 to 45 years, who were currently drug users or under treatment. In summary, after being trained by the research team, several substance user voluntaries helped the research team to find substance users and deliver a self-administered questionnaire to them. Also, an independent random sample from those who were under treatment in rehabilitation camps or other substance dependency treatment centers were selected. The sources of finding those withdrawing drug users were currently drug users who registered with private and public centers for treating drug dependency. The questionnaire was anonymous. As a result, the participants read the study consent but no signature was required. More details about the study procedures are provided before [12, 21].

### Selection of controls

As in many case-control studies [22–24], controls were selected from male hospital visitors (patients’ attendees or visitors) who visited the two city hospitals within few months of selecting the case group. To select control participants (non-substance users), adult (18–45 years of age) male visitors to the hospitals were asked to go to a pre-designed private room and read a letter describing the study aims and consent beginning with the following sentence: “Please pick up the questionnaire on the table and complete it if and only if you are not dependent to any substance; Otherwise please leave the room”. This is done because in Iran as many other similar countries, substance use disorder is a taboo and addicted people would not like to be known as so. The participants were provided with a refreshment pack in the room irrespective of filling up the questionnaire or not. Due to confidentiality and ethical considerations no attempt was made to confirm the self-reported status of the control participants [25, 26]. As the above process was completely anonymous, the control participants read the consent but no signature was required.

### Data collection

A self-administered questioner was made by the research group to collect data on the study variables among substance user participants before they started substance use. More information about constructing and validating the procedures are provided before [12]. Accordingly, the questionnaire was reasonably valid and reliable (Cronbach’s alpha = 0.65). The questionnaire was designed to collect information about the participant’s educational, social and behavioral related characteristics before substance use disorder in the case group and to the date of participation in the control group. All substance dependent individuals were in a private place when the questionnaire was delivered.

### Study variables

The questionnaire covered a wide range of areas including demographic (birth order, number of sisters and brothers, history of chronic disease, place of residency, level of education and history of failing during compulsory education), social (lost parents, parents were living together during childhood or adolescence, parents education and job,) and behavioral (criminal history, smoking status, smoking among relatives and friends, who would he talk with if needed during adolescent, most favorite leisure activities which the respondents preferred to do during adolescence, and aggressive behavior with other students at school). Again all questions regarded the time before which substance use was started.

### Inclusion criteria

Participants were all male aged between 18 to 45 and had minimum level of education (to be able to read and answer the questions). Both case and control groups were healthy enough to freely walk in to the centers or hospitals.

### Sample size and statistical analysis

A post hoc sample size calculation suggested that the sample size is adequate in order to detect an increase in the risk of substance use disorder as small as twice among primary educated participants compared to those with a college degree. The alpha value and power were set at 0.05 (two sided) and 80% respectively. To measure the adjusted association of each independent variable with the risk of substance use disorder, multiple logistic regression was applied. All variables were entered in to a multivariate logistic regression model using stepwise forward variable selection strategy. The modeling procedure was started after collinearity between the independent variables was measured using variance inflation factor index (VIF). The cut point for VIF was set at 10. SPSS version 19 is used for analysis of data.

## Results

The demographic and clinical characteristics of addicted and non-addicted participants are compared and presented in Table 1. Compared to the control group, participants in the case group had on average fewer sisters (2.75 ± 1.67 among addicted and 3.13 ± 1.82 among control participants, *p* = 0.01), had lower levels of education (57.80% of cases had not completed the compulsory education compared to 19.80% among controls, *p* < 0.001), failed during compulsory education (51.03% among cases compared to 33.17% among controls, *p* < 001), and had a history of smoking before substance use disorder (82.34% among cases compared to 15.58% among controls, *p* < 0.001).

**Table 1:** Baseline characteristics of the participants

Variables	Addicted (362)	Control (207)	*p*-value *
Quantitative measures	Mean±SD	Mean±SD	
Age	31.40±7.86	30.39±7.52	0.15
Age of losing parents	18.25±11.03	21.93±13.71	0.14
Birth order	3.30±2.14	3.92±2.59	0.002
Number of sisters	2.75±1.67	3.13±1.82	0.02
Number of brother	1.81±0.46	1.84±0.40	0.51
Age of starting cigarette	17.53±4.92	18.53±5.67	0.31
Qualitative measures	N(%)	N(%)	
Marital statues			
Single	154(43.63)	89(44.06)	0.92
Married	199(56.37)	113(55.94)	
Education			
Primary or secondary	200(57.80)	40(19.80)	<0.001
Diploma	103(29.77)	95(47.03)	
Collage	43(12.43)	67(33.17)	
Failed during compulsory education			
No	167(48.97)	135(66.83)	<0.001
Yes	174(51.03)	67(33.17)	
Argued with other students at school			
Always/Often	48(14.37)	12(6.35)	0.008
Sometimes	133(33.83)	82(43.39)	
Never	173(51.80)	95(50.26)	
Lost any of your family members			
No	241(73.48)	141(70.15)	0.40
Yes	87(26.52)	60(29.85)	
Who did you lose			
Father	42(51.85)	26(46.06)	0.90
Mother	29(35.80)	21(39.62)	
Brother/sister	10(12.35)	6(11.32)	
Father’s education			
Illiterate	75(26.13)	46(25.99)	0.99
Primary/ secondary	57(19.86)	36(20.34)	
High school/university	155(54.01)	95(53.67)	
Mother’s education			
Illiterate	62(22.63)	39(21.91)	0.94
Primary/ Secondary	39(14.23)	24(13.48)	
Diploma/university	173(63.14)	115(64.61)	
Mother’s job			
Homecare	301(95.86)	187(98.42)	0.11
Employed	13(4.14)	3(1.58)	
Father’s job			
Employee	73(23.55)	45(24.46)	0.10
Unemployed	25(8.06)	7(3.80)	
Self-employed	212(68.39)	132(71.74)	
Parents are living together			
No	38(11.73)	10(5.56)	0.02
Yes	286(88.27)	170(94.44)	
History of smoking^a^			
No	62(17.66)	168(84.42)	<0.001
Yes	289(82.34)	31(15.58)	
Smokers among relatives			
No	93(35.50)	11(37.93)	0.79
Yes	169(64.50)	18(62.07)	
Smokers among friends			
No	24(13.79)	3(17.65)	0.66
Yes	150(86.21)	14(82.35)	
Whom did you talk with at the time of having problem			
Family	219(64.99)	133(69.27)	0.04
Friends	97(28.78)	43(22.40)	
Nobody	13(3.86)	4(2.08)	
Others	8(2.37)	12(6.25)	
Leisure activities			
Home entertainment	23(8.19)	60(48.00)	<0.001
Outdoor entertainment	179(63.70)	53(42.40)	
Others	79(28.11)	12(9.60)	

*Mann-Whitney U or Chi-Squire test

^a^before addiction if was asked from addicted participants

After fitting the logistic model age, education, number of brothers, number of sisters, history of smoking, way of spending leisure time, failed during compulsory education and arguing with other students at school remained in the final model. Based on the results (Table 2), significant associations between number of sisters (OR = 0.82, 95% CI = 0.68 to 0.99, *P* = 0.04), history of smoking OR_yes/no_ = 19.89, 95%CI = 10.02 to 39.49, *P* < 0.001) and leisure time activities (OR_with friends/home_ = 8.98, 95%CI = 3.99 to 20.19, *P* < 0.001) were found with substance use disorder. On the other hand, no significant association between smoking or substance use disorder among family members, parents education or job and loss of family members with substance use was found (*P* > 0.05 for all).

**Table 2:** The association of study variables and addiction

Variable	OR addicted vs. control	CI95%	*P*-value
Number of sisters	0.82	0.68 to 0.99	0.04
History of smoking			
No	1.00	-	-
Yes	19.89	10.02 to 39.49	<0.001
Leisure activities			
Home entertainment	1.00	-	-
With friends	8.98	3.99 to 20.19	<0.001
Others	9.58	3.51 to 26.11	<0.001
Failed during compulsory education			
No	1.00	-	-
Yes	1.63	0.83 to 3.22	0.15
Argued with other students at school			
Never	1.00	-	-
Sometimes	1.31	0.66 to 2.63	0.43
Always/Often	1.75	0.55 to 5.48	0.33

## Discussion

This case-control study examined the value of a wide range of demographic, social and personal characteristics of the participants before they start using substance in the prediction of substance use disorder later in life.

The results suggested that participants with no or fewer sisters are more vulnerable to substance use disorder. In fact, it seems that sisters may protect their brothers from substance use or other risky behaviors. In that regard, both Slomkowski and Blanchard suggested that siblings have influence on the social and behavioral development of their sisters and brothers [27, 28]. In that regard, sisters are more sensitive and more responsive to family issues so that even elderlies feel closer to their sisters than brothers [29–31]. The relationship between sisters and brothers is also believed to take place depending on which sibling is older [32]. Sisters especially when they are older, have a maternal feeling to look after their younger brothers and sisters [33]. However, the function of family and the members’ interaction are of such complex context. For example, culture and religious background of family shapes the family relation and dynamics. As a result, the findings of the current study is to be considered with the significance of the above factors [34]. For example it has been suggested that culture and tradition play important roles in reduction of violence and abuse among family members [35]. It is therefore necessary to understand the effect of culture and tradition on the relationship between sisters and brothers, which seems to prevent them from substance use or other behavior disorders. The other significant finding suggested that addicted participants achieved less educational attainment and had more schooling problems. It is not clear from the results whether addiction is a cause of educational problems or addiction is affected by this important associated factor. Other studies suggested that individuals with less education are at higher risk of social and behavioral problems including substance use disorder. For example, Karrari [36] reported that lower levels of education is a powerful predictor of substance use. Based on the results of current study, substance user participants reported more failure during their compulsory education before starting to use substances (though the association was not statistically significant when it was adjusted for other study variables). This association has been addressed before by introducing commitment to school activities as a predictor of substance use disorder [37]. The results of current study also suggested a direct association between delinquency and later drug substance use. The association between criminal history and substance use is previously reported by Dark et.al. [38] as they suggested a direct and significant association between crime and heroin use. However, the association between criminal history and drug misuse seems to be bidirectional as it is suggested that substance use may also lead to criminal behavior [39, 40]. The importance of substance use in committing crime is reported by Green [41] who suggested that substance users are more prone to have criminal behaviors. Previous studies suggested that parental involvement in family practices helps family warmth and appropriate childhood conduction. On the other hand, Ineffective parental practices may cause childhood social and personal disorders and problems in family relationship and in turn educational achievement. This may drive children and adolescents to get involved in deviant peer groups making them vulnerable to chronic delinquent behaviors [42].

Among many factors associated with family relationship, parents’ socioeconomic status such as education is suggested to be particularly important [42]. However, in the present study, no significant association was found between parents’ educational status and substance use disorder among their children. This is in line with a report which was published by the US department of health on drug misuse in which, no or little association between parental education and substance use among their children was reported. This may suggest that preventing substance use disorder needs especial parental skills which are not necessarily obtained by higher education. Despite this finding, the results from the first phase of the current study suggested that withdrawing attempt is inversely associated with the level of education of the fathers of substance users [43]. The present study also introduced the importance of the ways a young person spends his leisure time in the risk of becoming a substance user. Accordingly individuals who spent their leisure time with their family, have lower risk of becoming substance user later in their life. No published study on similar subject was found. The results of a study conducted by Kwan [44] suggested that exercise at either home or outdoor is a protective factor for substance use.

Finally, as the strongest risk factor, history of smoking (before substance use was started) was associated with a higher chance of substance use. This finding was supported by another study [45].

## Conclusion

The results of current study revealed smoking and leisure time as the most powerful predictive factors. Smoking has been previously known as a common miss behavior among substance users. The results of current study suggested that smoking is actually a major predictive factor, which can be started years before becoming a substance user or even first substance use. This finding can be used to detect high-risk adolescents and prevent them from becoming a drug user. The way adolescents spend their leisure time especially when they are spending their time outside home with friends is also a valuable predictive factor that, if well understood, can be used by parents to do better supervision and prevent young members of family to acquire miss-behaviors including early smoking and drug use. As a result, warm family environment via reducing time spent with friends is potentially an important factor in preventing under-age smoking and substance use in later life. Although the preventive effect of having more sisters needs more investigation to explain the mechanism of action, it possibly suggests the importance of family members especially sisters in preventing substance use. The results suggested that parents need to provide their children with warmer family environment as home or provide them with family leisure times. These findings can be used for early detection of at risk adolescents by teachers or parents.

### Limitations

Substance use is a crime and socially condemned behavior in Iran. Therefore, aside from self-reporting, no test or independent source of information was available to confirm the actual status of the participants. This issue is not expected to have a serious effect on the results, as a person with no substance use hardly introduce himself as drug user and considering the estimated prevalence of substance use in Iran (1.6%), even if the control participants falsely introduce themselves as non-user the results would not be affected significantly. Neither cases nor controls were selected randomly from their relevant populations. This is because substance use disorder is illegal and substance users are mosetly un-known and hard to reach. As a result, a private place was needed to encourage potential controls reveal their actual substance use status. Accordingly, snowball sampling and self-reported information was the only feasible option available. This approach is quite common in studies on special groups [46].

## Abbreviation

VIF: Variance inflation factor index
